# Human endonuclease V is a ribonuclease specific for inosine-containing RNA

**DOI:** 10.1038/ncomms3273

**Published:** 2013-08-05

**Authors:** Yoko Morita, Toshihiro Shibutani, Nozomi Nakanishi, Kazuko Nishikura, Shigenori Iwai, Isao Kuraoka

**Affiliations:** 1 Graduate School of Engineering Science, Osaka University, 1-3 Machikaneyama, Toyonaka, Osaka 560-8531, Japan; 2 The Wistar Institute, 3601 Spruce Street, Philadelphia, Pennsylvania 19104, USA

## Abstract

Deamination of DNA bases can create missense mutations predisposing humans to cancer and also interfere with other basic molecular genetic processes; this deamination generates deoxyinosine from deoxyadenosine. In *Escherichia coli*, the highly conserved endonuclease V is involved in alternative excision repair that removes deoxyinosine from DNA. However, its exact activities and roles in humans are unknown. Here we characterize the FLJ35220 protein, the human homologue of *E. coli* endonuclease V, hEndoV as a ribonuclease specific for inosine-containing RNA. hEndoV preferentially binds to RNA and efficiently hydrolyses the second phosphodiester bond located 3′ to the inosine in unpaired inosine-containing ssRNA regions in dsRNA. It localizes to the cytoplasm in cells. The ribonuclease activity is promoted by Tudor staphylococcal nuclease and detected on inosine-containing dsRNA created by the action of adenosine deaminases acting on RNA. These results demonstrate that hEndoV controls the fate of inosine-containing RNA in humans.

## Abstract (web summary)

In *Escherichia coli*, the highly conserved enzyme endonuclease V has a role in DNA repair. Here the authors show that human endonuclease V is an inosine 3' endoribonuclease and that Tudor Staphylococcal nuclease enhances this activity, suggesting a role for human endonuclease V in RNA metabolism.

## Graphical abstract

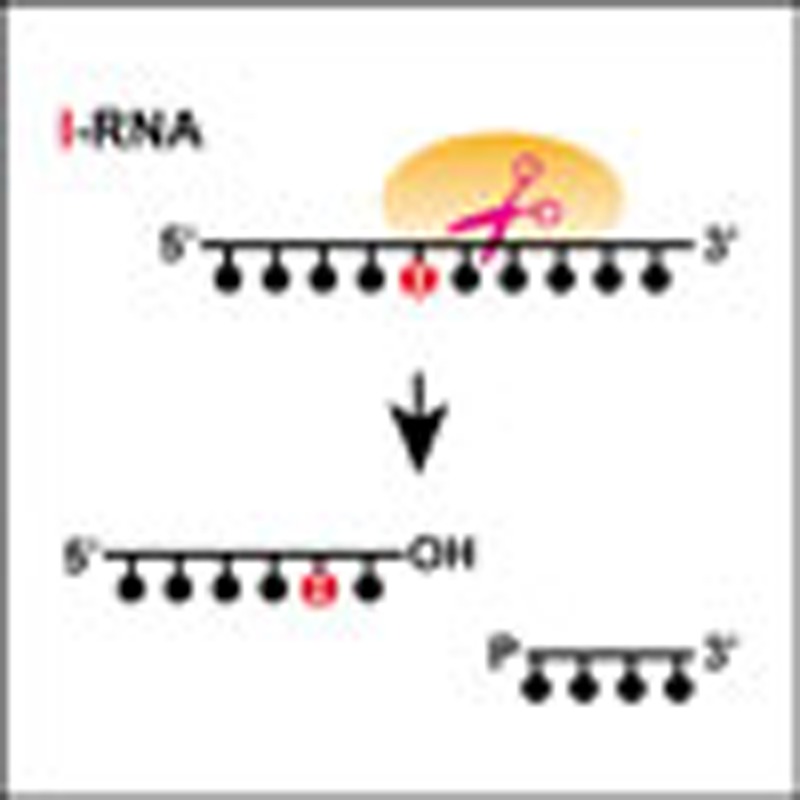

Although genomic DNA contains genetic information that should be error free to facilitate the proper functioning of the cell, DNA is prone to deterioration and modifications due to environmental and endogenous damage. Under physiological conditions, deamination of DNA bases can occur spontaneously and at a physiologically significant rate via a hydrolytic reaction. Three of the four DNA bases (cytosine, adenine and guanine) have amino groups. Deamination of these three bases generates the base analogues uracil, hypoxanthine and xanthine, respectively^1^. Bases in DNA and nucleotides can be deaminated both chemically and enzymatically. Deamination of DNA precursors can lead to the accumulation of noncanonical nucleoside triphosphates in the cell’s nucleotide pool^2^,^3^. These noncanonical nucleotides, such as dITP, dXTP and dUTP, can be incorporated into newly synthesized DNA, albeit less efficiently than regular DNA precursors. As deaminated bases in DNA have pairing specificities different from the original bases, these lesions have high mutagenic potential and may contribute to the incidence of genetic diseases and cancer. Because of these risks, cells possess excision repair functions to prevent mutagenesis^4^.

Excision repair systems are highly conserved from bacteria to humans^4^. There are several major DNA repair pathways, such as nucleotide excision repair (NER), which operates primarily on bulky helix-distorting damage caused by ultraviolet irradiation or genotoxic chemicals, and base excision repair (BER), which is used for nonbulky and non–helix-distorting DNA modifications induced by alkylation, oxidation and deamination. Deoxyuridine is a nucleoside formed when uracil is attached to a deoxyribose ring; this deamination product is subjected to BER, which is initiated by a uracil-DNA glycosylase that searches the genome to locate the sites of damage and then catalyses the hydrolysis of the N-glycosidic bond to release the lesion and generate an apurinic/apyrimidinic (AP) site. Downstream enzymes subsequently process this AP site and use the information from the undamaged strand to restore the original coding sequence^5^.

Deoxyinosine is a nucleoside formed when hypoxanthine is attached to a deoxyribose ring. Although deoxyinosine is repaired by alkyladenine DNA glycosylase (AAG) in BER, it is also subjected to alternative excision repair (AER), which is biochemically distinct from NER and BER^6^,^7^,^8^. While NER involves the dual excision of the damaged flanking sites and BER involves the cleavage of the N-glycosidic bond between the damaged base and the sugar in DNA, this AER pathway involves the initiation of a single nick, strictly on one side of the base damage in the DNA strand. The AER that has been most extensively defined by studies in *E. coli* involves a specific endonuclease called endonuclease V (*E. coli* endonuclease V: eEndoV), a product of the *nfi* gene in *E. coli*^9^. Studies on the mutant strain have revealed a high frequency of A:T to G:C transitions following exposure to nitrous acid, which induces deoxyinosine in DNA^10^,^11^. Deoxyinosine has strong miscoding properties because it pairs with C. Purified eEndoV is a deoxyinosine 3′ endonuclease that recognizes both double-stranded and single-stranded DNAs (dsDNA and ssDNA, respectively) containing deoxyinosine and cleaves the second and third phosphodiester bonds 3′ to the mismatch of deoxyinosine, leaving a nick with 3′ hydroxyl and 5′ phosphate groups. Moreover, this enzyme has subsequently been shown to have a broad substrate spectrum and acts at AP sites, urea residues, base mismatches, flap DNA, pseudo Y structures and loops, and hairpins^6^,^12^,^13^. As it cannot release deoxyinosine or the damaged bases, eEndoV is thought to require another enzyme to completely repair the DNA containing deoxyinosine^8^,^14^. In *E. coli* and some other bacteria, there is an eEndoV-dependent AER pathway for the removal of inosine from DNA. In mammalian cells, hEndoV and mouse EndoV (mEndoV) have been proposed to have a similar role^8^,^15^. However, the AER pathway has not yet been elucidated in mammalian cells, and substantial discrepancies have been reported in the biochemical results of mammalian EndoV. Purified hEndoV from *E. coli* recognizes and cleaves both dsDNA and ssDNA containing deoxyinosine, by using nanomolar concentrations of the fluorescent substrate containing deoxyinosine^15^. Specific activity has been observed for the mEndoV in ssDNA containing deoxyinosine, but this activity is very poor in dsDNA containing deoxyinosine^8^. On the other hand, Fladeby *et al.*^16^ showed that hEndoV neither cleaved nor bound to dsDNA substrates containing deoxyinosine under the same conditions in which eEndoV cleaved efficiently. Moreover, mEndoV cleaves ssDNA substrates but has reduced activity for dsDNA substrates^8^.

Here we purified recombinant hEndoV and characterized the function of this deoxyinosine 3′ endonuclease. Our findings indicate that hEndoV prefers RNA substrates over DNA substrates. Deoxyinosine is found in DNA, while inosine is found in RNA due to spontaneous or nitrosative deamination reactions and the incorporation of inosine triphosphate (ITP) during transcription^2^,^3^. Inosine has been implicated in RNA editing^17^,^18^,^19^,^20^, a post-transcriptional modification that alters the sequence of RNA from the original sequence encoded in the DNA. Interestingly, RNA from brain tissue is the most frequently edited^21^. Thus, as hEndoV can cleave inosine-containing RNA (i-RNA) at specific sites, this RNA-editing activity may be a key factor determining the fate of i-RNA.

## Results

### hEndoV amino-acid sequence

Five representative EndoV homologues were aligned to examine similarities between bacteria and mammals (Supplementary Fig. S1). A complementary DNA (cDNA) sequence for FLJ35220 (hEndoV), exhibiting homology to eEndoV, was cloned from total human dermal fibroblast RNA. The encoded open reading frame predicted a product of 282 amino-acid residues, with a calculated molecular mass of 31 kDa. Alignment of the amino-acid sequence with that of eEndoV revealed the following characteristics for hEndoV and eEndoV: identities=74/199 (37%), positives=108/199 (54%) and gaps=19/199 (10%). The predicted active site in hEndoV (Asp52, Tyr91 or Glu100) was conserved from *E. coli* to humans, corresponding to Asp43, Tyr80 or Glu89 in eEndoV, that is, sites that had a significant effect on catalysis in *Thermotoga maritima*^22^,^23^.

### Properties of hEndoV

**Figure 1:**
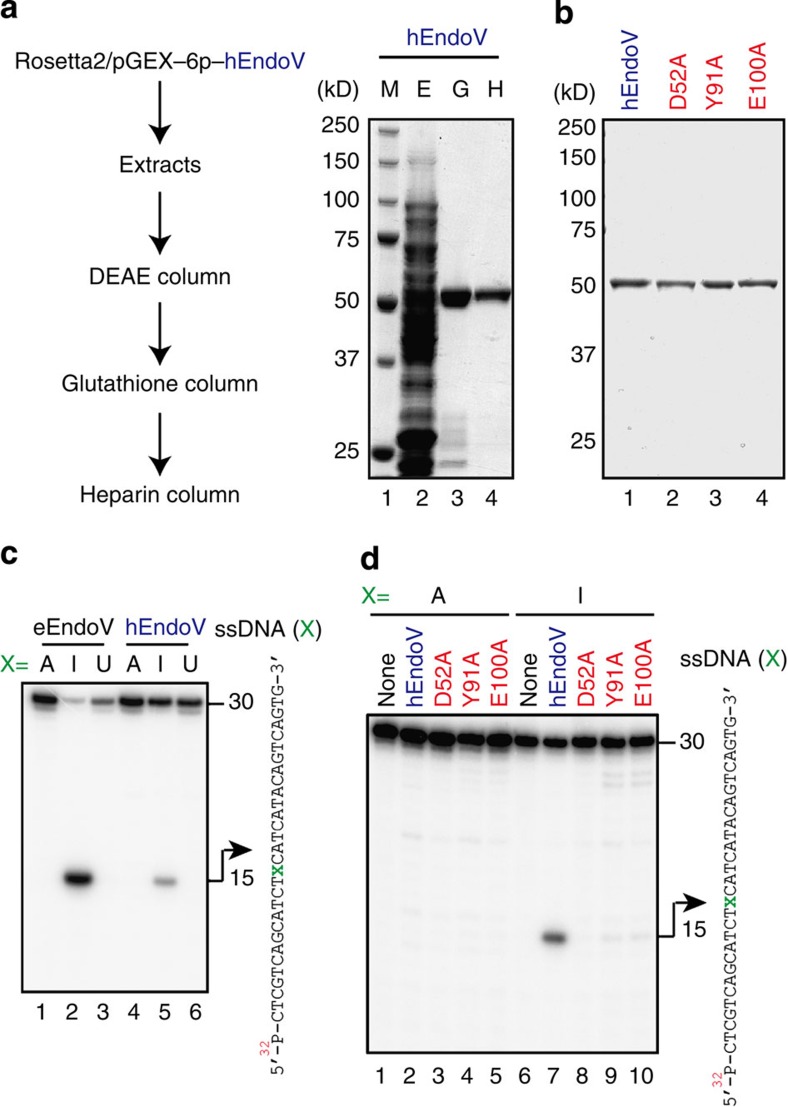
Deoxyinosine 3′ endonuclease activity of recombinant hEndoV. (**a**) Purification of recombinant hEndoV. Experimental procedure for purification of hEndoV. Aliquots from various steps of the purification were subjected to SDS–polyacrylamide gel electrophoresis (SDS–PAGE) on 10% gels, and the proteins were visualized by staining with Coomassie Brilliant Blue (CBB). Lane 1, marker; lane 2, extracts (E); lane 3, glutathione column eluate (G); lane 4, heparin column eluate (H). (**b**) Mutant proteins were subjected to SDS–PAGE on 10% gels and were visualized by staining with CBB. Lane 1, hEndoV; lane 2, D52A; lane 3, Y91A; lane 4, E100A. (**c**) ^32^P-labelled 30-mer ssDNA containing deoxyadenosine (lanes 1 and 4), deoxyinosine (lanes 2 and 5) or deoxyuridine (lanes 3 and 6) in position X (right panel) was incubated with eEndoV (5 nM; lanes 1–3) or hEndoV (300 nM; lanes 4–6). A 30-mer ssDNA (right panel) was cleaved at the indicated positions (arrows, 15-mer). (**d**) ^32^P-labelled 30-mer ssDNA containing deoxyadenosine (lanes 1–5) or deoxyinosine (lanes 6–10) was incubated with a series of hEndoV mutant proteins (300 nM): hEndoV, lanes 2 and 7; D52A, lanes 3 and 8; Y91A, lanes 4 and 9; and E100A, lanes 5 and 10. A 30-mer ssDNA (right panel) was cleaved at the indicated positions (arrows, 15-mer).

To examine the biological activities of the protein encoded by the *hEndoV* cDNA, we prepared recombinant hEndoV with a glutathione *S*-transferase (GST) N-terminal tag in *E. coli* and purified the recombinant protein (Fig. 1a). As expected from the calculated relative molecular mass, purified hEndoV bearing the GST-tag migrated as a single band of approximately 55 kDa in SDS–polyacrylamide gel electrophoresis analysis (Fig. 1a). Mutant proteins bearing alanine substitutions at Asp52, Tyr91 or Glu100 in hEndoV were purified as a single band by the same procedures (Fig. 1b).

To study the ability of hEndoV to cleave the second and third phosphodiester bonds 3′ to the deoxyinosine, which is known as deoxyinosine 3′ endonuclease activity in eEndoV, a 30-mer ssDNA oligonucleotide was synthesized containing deoxyinosine residue 14 (Fig. 1c). hEndoV, like eEndoV, could cleave ssDNA containing a deoxyinosine but was inefficient at cleaving ssDNA containing deoxyadenosine or deoxyuridine, which are cytosine deamination products (Fig. 1c). However, although hEndoV could cleave ssDNA containing deoxyinosine, its activity was low (see Fig. 1c, lanes 2 and 5). All mutations, that is, D52A, Y91A and E100A, of the predicted active site resulted in proteins that could not show the inherent nuclease activity in the ssDNA substrate containing deoxyinosine under our experimental conditions, although Y91A and E100A did show a very low level of activity (Fig. 1d).

Next, we tested whether hEndoV could cleave dsDNA substrates containing deoxyinosine. While both eEndoV and hEndoV could cleave ssDNA substrates containing deoxyinosine (Supplementary Fig. S2), differential cleavage functions were observed for dsDNA substrates. In dsDNA substrates containing deoxyinosine paired with either deoxythymine (dI:dT) or deoxycytosine (dI:dC), eEndoV exhibited cleavage activity; however, hEndoV had very little activity towards both of these substrates (Supplementary Fig. S2), suggesting that hEndoV required regions of ssDNA within the dsDNA for cleavage of the second and third phosphodiester bonds 3′ to the deoxyinosine. Interestingly, despite this inability to efficiently cleave dsDNA containing dI:dT or dI:dC, hEndoV had an affinity for ssDNA/dsDNA containing deoxyinosine (Supplementary Fig. S2) and for dsDNA containing no deoxyinosine (Supplementary Fig. S3). Moreover, as eEndoV appeared to have a broad substrate specificity, we tested whether hEndoV cleaved DNA-damaged substrates containing AP sites, *O*^*6*^-methylguanine, 8-oxoguanine, 5-methylcytosine, *cis*-syn cyclobutane pyrimidine dimers or pyrimidine(6-4)pyrimidone photoproducts. However, we did not observe these nuclease activities of hEndoV with these ssDNA substrates (Supplementary Fig. S4).

**Figure 2:**
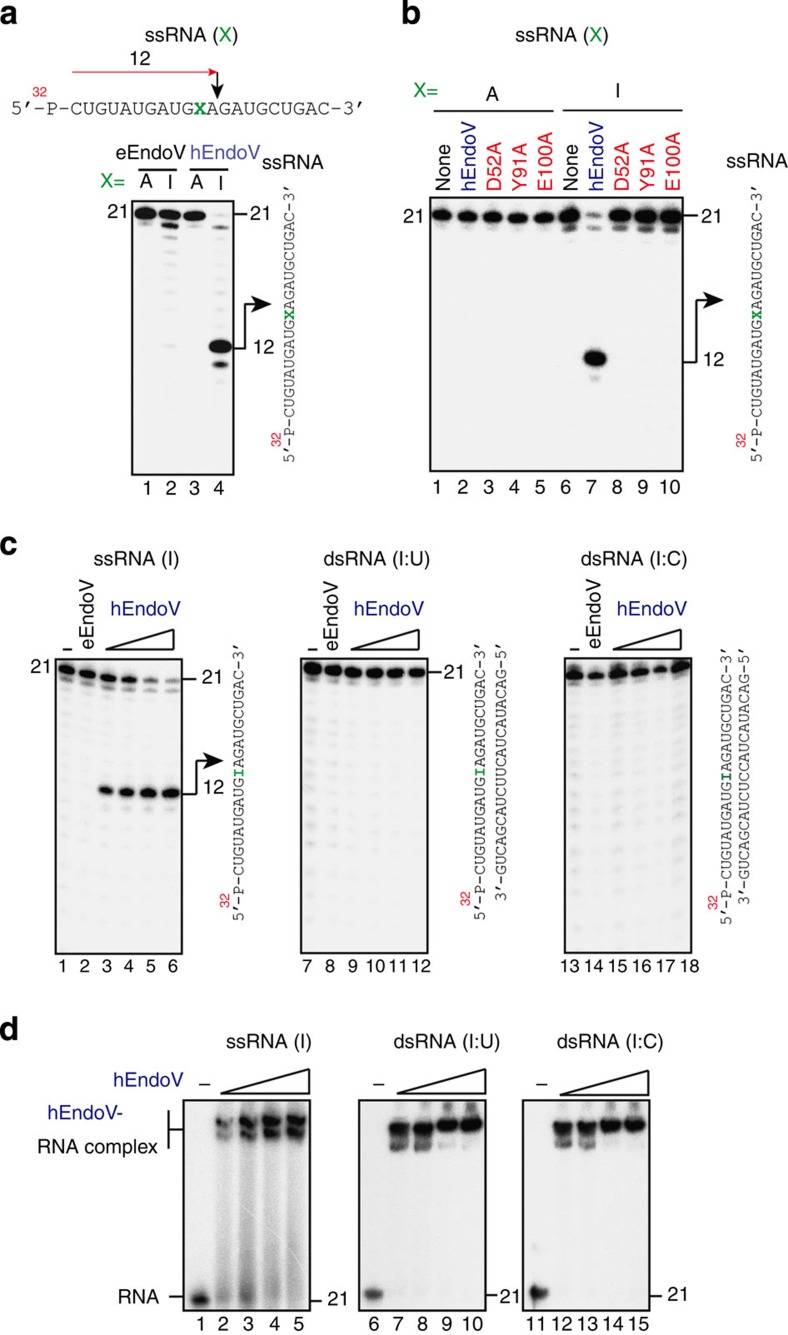
Properties of hEndoV for inosine-containing RNA. (**a**) ^32^P-labelled 21-mer ssRNA containing adenosine (lanes 1 and 3) or inosine (lanes 2 and 4) in position X (right panel) was incubated with eEndoV (5 nM; lanes 1 and 2) or hEndoV (30 nM; lanes 3 and 4). A 21-mer ssRNA (right panel) was cleaved at the indicated positions (arrows, 12-mer). (**b**) ^32^ P-labelled 21-mer ssRNA containing adenosine (lanes 1–5) or inosine (lanes 6–10) was incubated with a series of hEndoV mutant proteins (30 nM): hEndoV, lanes 2 and 7; D52A, lanes 3 and 8; Y91A, lanes 4 and 9; and E100A, lanes 5 and 10. A 21-mer ssRNA (right panel) was cleaved at the indicated positions (arrows, 12-mer). (**c**) ^32^ P-labelled 21-mer ssRNA containing inosine (lanes 1–6), dsRNA containing inosine paired with uridine (lanes 6–12) or dsRNA containing inosine paired with cytidine (lanes 13–18) was incubated with increasing concentrations of hEndoV (1, 2, 4 and 8 nM in each group of 4 lanes) or eEndoV (5 nM). A 21-mer ssRNA or dsRNA (right panel in each figure) was cleaved at the indicated positions (arrows, 15-mer). (**d**) ^32^ P-labelled 21-mer ssRNA or dsRNA shown in **c** was incubated at 4 °C for 30 min with increasing concentrations of hEndoV (0, 12.5, 25, 50 and 100 nM in each group of 5 lanes). Free and bound fractions were separated on a nondenaturing 8% polyacrylamide gel.

The deamination reaction occurs not only in DNA but also in RNA, and inosine can be found in RNA due to the activities of RNA-editing enzymes^18^ or by incorporation of ITP during transcription^2^,^3^. Thus, we next asked whether hEndoV-mediated cleavage could occur in RNA containing inosine. A 21-mer RNA oligonucleotide containing an inosine 11 bp from the 5′ terminus was synthesized (Fig. 2a). Incubation of eEndoV with ssRNA containing inosine revealed no significant formation of cleavage products (Fig. 2a). However, hEndoV was able to generate a labelled 12-mer, indicating that the main cleavage sites were on the second and third phosphodiester bonds 3′ to the inosine (Fig. 2a and Supplementary Fig. S5). All mutated proteins, that is, those including D52A, Y91A and E100A point mutations, were unable to efficiently cleave the ssRNA substrate containing inosine (Fig. 2b). We then tested whether hEndoV could cleave a dsRNA substrate containing inosine. While hEndoV could cleave ssRNA substrates to generate labelled 12-mers in a protein concentration-dependent manner, neither enzyme could generate any specific products in reactions with dsRNA containing I:U or I:C pairings (Fig. 2c). However, hEndoV had an affinity for both ssRNA and dsRNA containing inosine (Fig. 2d). Furthermore, similar to the affinity for dsDNA, this enzyme also had an affinity for dsRNA containing no inosine, indicating that inosine in dsRNA is dispensable with respect to the dsRNA binding of hEndoV (Supplementary Fig. S6).

### RNA is preferred to DNA as a substrate by hEndoV

**Figure 3:**
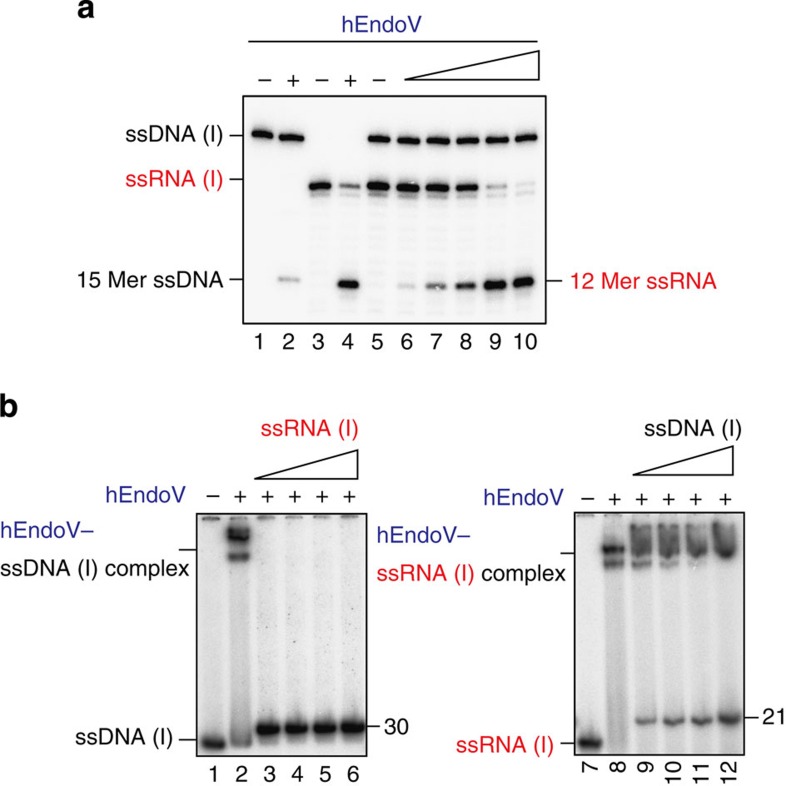
RNA is preferred to DNA by hEndoV as a substrate. (**a**) ^32^ P-labelled 30-mer ssDNA containing deoxyinosine and/or 21-mer ssRNA containing inosine was incubated with hEndoV (lanes 1, 3 and 5: 0 nM; lane 2: 150 nM; lane 4: 8 nM; and lanes 6–10: 1, 2, 4, 8 and 16 nM). Cleavage sites were analysed by electrophoresis. (**b**) hEndoV preferentially binds to ssRNA rather than ssDNA. ^32^P-labelled 30-mer ssDNA containing deoxyinosine was incubated with hEndoV (lane 1: 0 nM and lanes 2–6: 300 nM) and cold ssRNA containing inosine (lanes 2–6: 0, 2.5, 5.0, 7.5 and 10 μM; left panel). ^32^ P-labelled 21-mer ssRNA containing inosine was incubated with hEndoV (lane 7: 0 nM and lanes 8–12: 300 nM) and cold ssDNA containing deoxyinosine (lanes 8–12: 0, 2.5, 5.0, 7.5 and 10 μM; right panel). Free and bound fractions were separated on a nondenaturing 8% polyacrylamide gel.

As our data indicated that hEndoV could cleave ssDNA substrates containing deoxyinosine and ssRNA substrates containing inosine, we next asked which substrates, ssDNA or ssRNA, were preferred by hEndoV. Thirty-mer ssDNA substrates containing deoxyinosine and 21-mer ssRNA substrates containing inosine were simultaneously incubated with hEndoV. As shown in Fig. 3a, the enzyme could cleave ssRNA to generate 12-mer-labelled RNA products in a protein concentration-dependent manner, but was inefficient at ssDNA cleavage (Fig. 3a). On the other hand, the enzyme was able to cleave both ssDNA and ssRNA substrates when either substrate was used alone (Fig. 3a), albeit with lower efficiency in assays using ssDNA. In addition, we examined DNA/RNA competition binding assays for hEndoV. When the enzyme was incubated with ssDNA, protein–DNA complexes were observed (Fig. 3b); incubation with ssRNA containing inosine as binding competitors caused these complexes to disappear (Fig. 3b). On the other hand, hEndoV–RNA complexes were observed, even during incubation with ssDNA competitors containing deoxyinosine (Fig. 3b). Therefore, these data suggested that hEndoV enzymatically preferred ssRNA substrates to ssDNA substrates.

### hEndoV is a cytoplasmic enzyme

**Figure 4:**
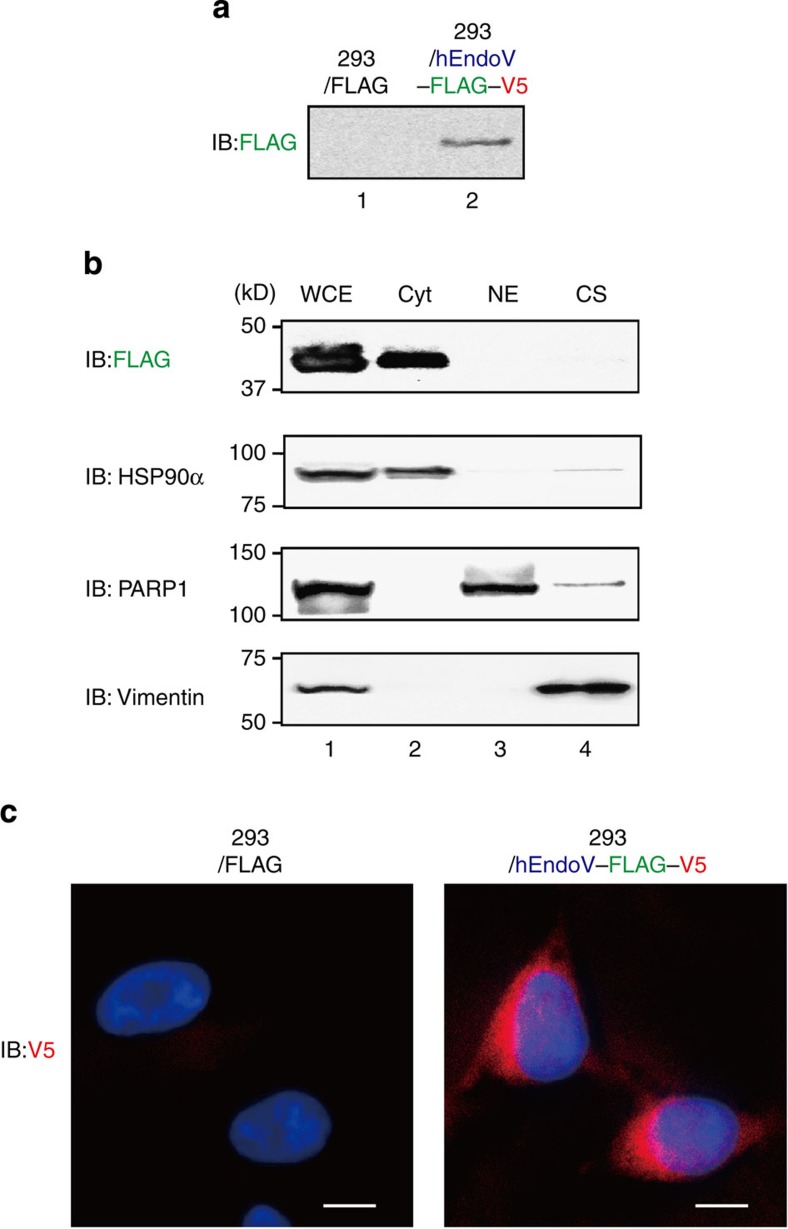
Localization of hEndoV *in vivo*. (**a**) FLAG-V5-expressing HEK293 cells (lane 1) or hEndoV-FLAG-V5-expressing HEK293 cells (lane 2) were established, and the hEndoV-FLAG-V5 was detected with anti-FLAG antibodies. (**b**) Whole cell (WCE: lane 1), cytoplasmic (Cyt: lane 2), nuclear (NE: lane 3) and cytoskeletal (lane 4) extracts were detected with anti-FLAG antibodies. The purity of these fractions was confirmed with antibodies against HSP90α, PARP1 and vimentin. (**c**) The expression of hEndoV in HEK293 cells was detected with anti-V5 antibodies and visualized with Alexa Fluor 568-conjugated anti-mouse IgG antibodies (red) and 4',6-diamidino-2-phenylindole dihydrochloride (blue). FLAG-V5-expressing HEK293 cells (left) and hEndoV-FLAG-V5-expressing HEK293 cells (right) are shown. Scale bars, 10 μM.

Next, to determine the subcellular localization of hEndoV, we established an HEK293 cell line stably expressing a C-terminally FLAG-V5-6xHis-tagged hEndoV (hEndoV–FVH; Fig. 4a). The cells were lysed and fractionated into cytosolic, nuclear and cytoskeletal fractions, and the hEndoV–FVH and representative proteins (HSP90α, PARP1 and vimentin) in each fraction were analysed by immunoblotting. All indicated proteins were detected in whole cell extracts (Fig. 4b). In contrast, using an antibody designed to recognize the C-terminal tag, hEndoV–FVH was found predominantly in the cytosolic fraction, corresponding to the fraction containing HSP90α (Fig. 4b), but was not detected in nuclear or cytoskeletal fractions (Fig. 4b). In addition, immunofluorescence was also used to examine the localization of hEndoV in hEndoV–FVH-expressing HEK293 cells. hEndoV–FVH was found predominantly in the cytoplasm, but no significant signals were detected in control HEK293 cells (Fig. 4c). Additionally, the subcellular localization of hEndoV was predicted by the WoLF PsoRT. These results suggested that hEndoV acts in the cytoplasm, indicating cytoplasmic RNA, but not genomic DNA, as its likely substrate.

### hEndoV is a structure-specific inosine-containing dsRNase

**Figure 5:**
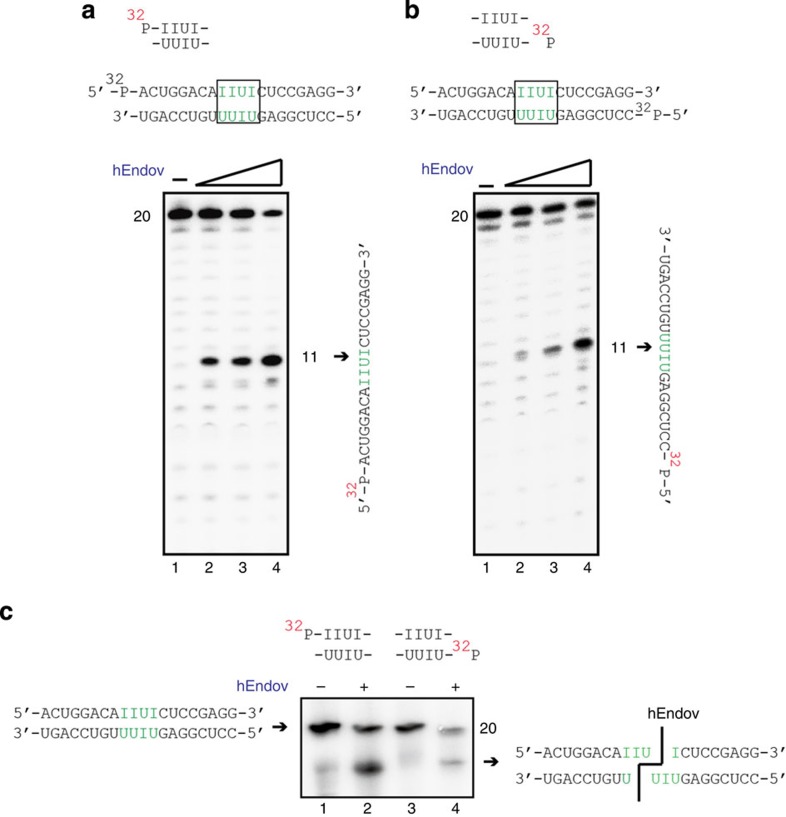
hEndoV is a ribonuclease specific for inosine-containing dsRNA. (**a**) ^32^ P-labelled 20-mer dsRNA containing a 5′-IIUI-3′-specific sequence (top panel) was incubated with hEndoV (0, 1, 2 and 4 nM in each group of 4 lanes). A 20-mer ssRNA (right panel) was cleaved at the indicated positions (arrows, 11 mer). (**b**) ^32^ P-labelled 20-mer dsRNA containing a 5′-UUIU-3′-specific sequence (top panel) was incubated with hEndoV (0, 1, 2 and 4 nM in each group of 4 lanes). A 20-mer ssRNA (right panel) was cleaved at the indicated positions (arrows, 11-mer). (**c**) ^32^ P-labelled 20-mer dsRNA containing a 5′-IIUI-3′- (**a**: top panel, lanes 1 and 2) and 5′-UUIU-3′- (**b**: top panel, lanes 3 and 4) specific sequence was incubated with hEndoV (lanes 1 and 3: 0 nM; lanes 2 and 4: 4 nM). The mixtures were separated on a nondenaturing 12% polyacrylamide gel.

The ribonuclease activity that preferentially cleaves inosine-containing dsRNA at 5′-IIUI-3′ and 5′-UIUU-3′ was previously identified in various mammalian cell extracts^24^,^25^; however, the enzyme responsible for this activity had not yet been identified. hEndoV could cleave ssRNA substrates containing inosine but not dsRNA substrates containing inosine (Fig. 2c). Thus, we determined whether hEndoV preferentially cleaved specific sites (that is, 5′-IIUI-3′) containing 20-mer dsRNA oligonucleotides, unlike the inosine sites we used that contained 21-mer dsRNA oligonucleotides. We found that hEndoV indeed cleaved IIUI substrates, generating 11-mer-labelled ssRNA substrates in an hEndoV concentration-dependent manner (Fig. 5a). Using the 5′-labelled complementary ssRNA strand containing inosine (5′-UIUU-3′; Fig. 5b), we found that hEndoV could cleave these UIUU substrates, generating 11-mer-labelled ssRNA substrates (Fig. 5b). Next, we investigated whether hEndoV, acting as a dsRNA-specific endoribonuclease RNaseIII, could generate two short dsRNA fragments from one 20-mer dsRNA fragment containing inosine. Each reaction mixture was separated on a nondenaturing gel. We found that hEndoV could produce two short dsRNA fragments, indicating that hEndoV was capable of simultaneously cleaving dsRNA fragments containing the specific sites 5′-IIUI-3′ and 5′-UIUU-3′ (Fig. 5c).

**Figure 6:**
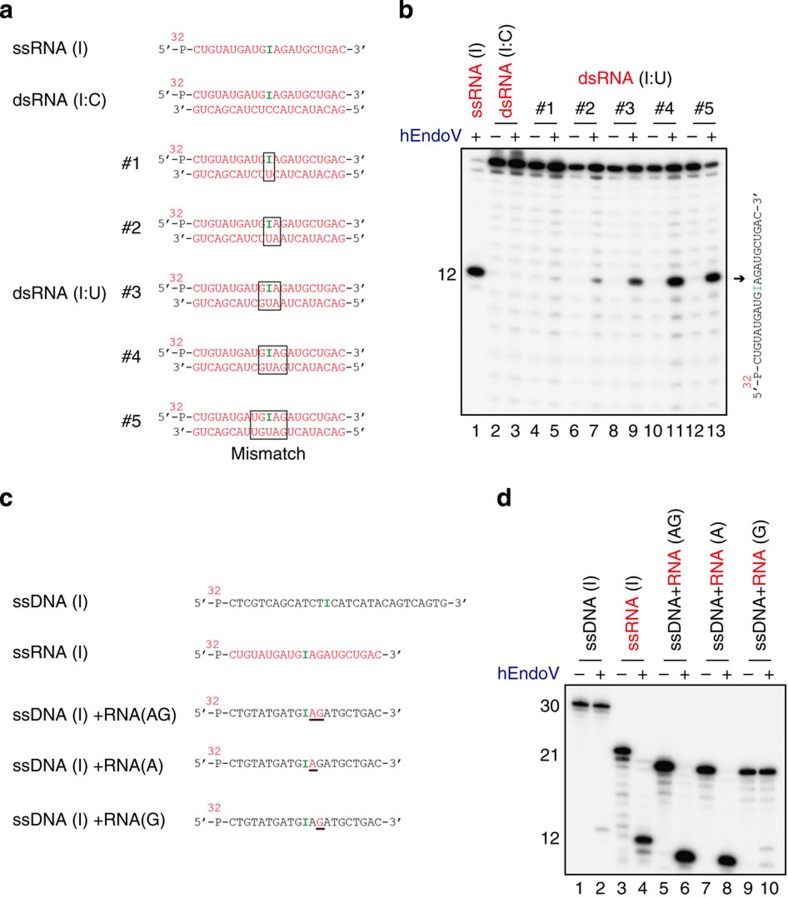
hEndoV is a structure-specific inosine-containing dsRNase. (**a**) RNA substrates containing inosines within a series of mismatch regions included ssRNA (I), dsRNA (I/C) and dsRNA (I/U). Each substrate was 5′-labelled on the top strand. (**b**) ^32^P-labelled 21-mer ssRNA or dsRNA was incubated with hEndoV (8 nM) in odd-numbered lanes. A 21-mer ssRNA (right panel) was cleaved at the indicated positions (arrows, 12-mer). (**c**) ^32^P-labelled substrates containing inosine or deoxyinosine are indicated as ssDNA (I), ssRNA (I), ssDNA (I)+RNA (AG), ssDNA (I)+RNA (A) and ssDNA (I)+RNA (G). The underlines in ssDNA (I)+RNA (AG), ssDNA (I)+RNA (A) and ssDNA (I)+RNA (G) indicate RNA. (**d**) ^32^P-labelled substrates shown in **c** were incubated with hEndoV (8 nM). Cleavage sites were analysed by electrophoresis.

Although hEndoV exhibited very low efficiency towards cleavage of dsRNA substrates containing inosine, this enzyme could cleave dsRNA substrates containing the specific sites 5′-IIUI-3′ and 5′-UIUU-3′. Using dsRNA substrates containing a series of mismatch pairs (Fig. 6a), we investigated which dsRNA structures were required by hEndoV. Cleavage of ssRNA containing inosine in dsRNA substrates was observed in a mismatched pair-dependent manner (Fig. 6b) but not in I:C or I:U pairs. The efficiency reached a plateau at four mismatched base pairs, indicating that hEndoV requires inosine containing-local distortions, which are induced by mismatch pairs or wobble base pairs, in dsRNA substrates. This enzyme could bind to ssDNA, dsDNA, ssRNA and dsRNA containing hypoxanthine (Supplementary Fig. S2 and Fig. 2d). However, ribonuclease activity was observed mainly in ssRNA substrates and in dsRNA substrates containing-local distortions, but not in DNA substrates. These findings suggested that the activity requires a hydroxyl group at the 2′ position of ribose. To test this, ssDNA oligonucleotide substrates containing either second and/or third riboses 3′ to the deoxyinosine (Fig. 6c) were examined in nuclease assays. As expected, cleavage by hEndoV was observed in ssDNA substrates containing second and third riboses 3′ to deoxyinosine, similar to ssRNA substrates containing inosine (Fig. 6d). In the second ribose, hEndoV could cleave ssRNA substrates containing inosine; however, this cleavage was not observed in the third ribose, indicating that the activity required the hydroxyl group of the second ribose 3′ to the hypoxanthine.

### hEndoV is an I-RNase

**Figure 7:**
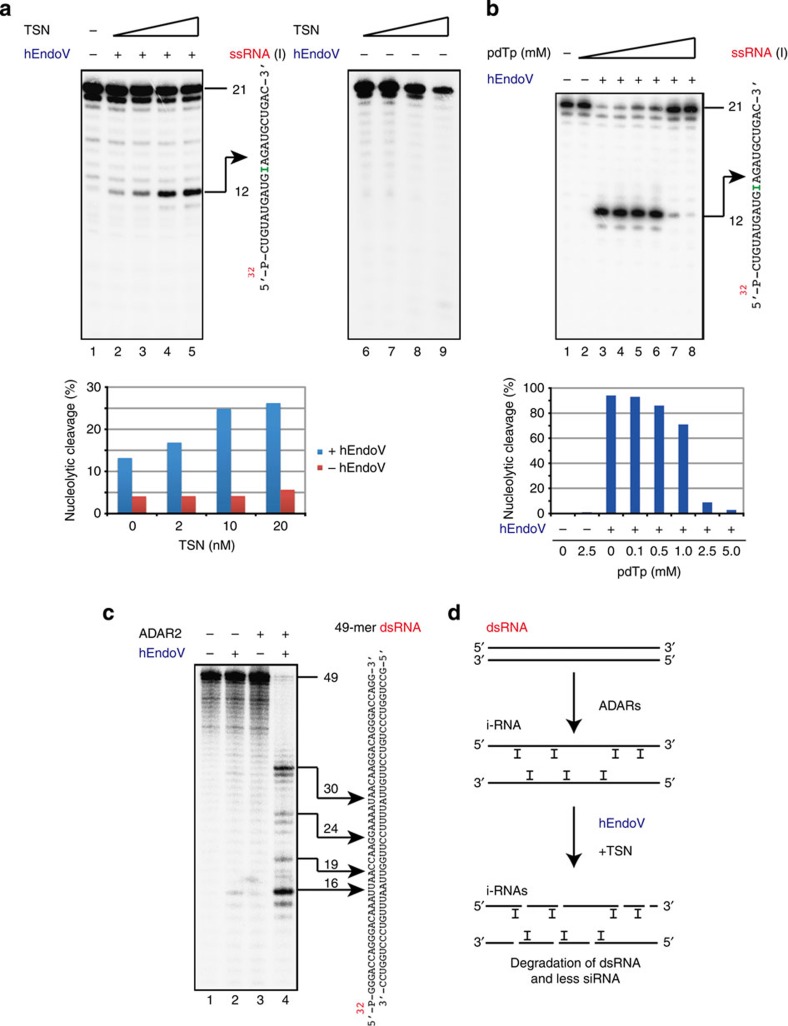
hEndoV cleaved dsRNA as an I-RNase with TSN and ADAR2. (**a**) ^32^P-labelled 21-mer ssRNA containing inosine was incubated with hEndoV (lanes 2–5: 4 nM; lanes 1 and 6–9: 0 nM) and TSN (lanes 2–5 and 6–9: 0, 2, 10 and 20 nM). Cleavage sites were analysed by electrophoresis. A 21-mer ssRNA (right panel) was cleaved at the indicated positions (arrows, 12 mer). The graph showing yields of 12-mer products with increasing TSN concentration. (**b**) ^32^ P-labelled 21-mer ssRNA containing inosine was incubated with hEndoV (4 nM) and pdTp (lanes 2–8: 2.5, 0, 0.1, 0.5, 1, 2.5 and 5 mM). A 21-mer ssRNA (right panel) was cleaved at the indicated positions (arrows, 12-mer). The graph showing yields of 12-mer products with increasing pdTp concentration. (**c**) ^32^ P-labelled 49-mer dsRNA (lane 1) was incubated with hEndoV (lanes 2 and 4: 4 nM) and ADAR2 (lanes 3 and 4: 1 nM). Cleavage sites were analysed by electrophoresis. A 49-mer dsRNA (right panel) was cleaved at the indicated positions (arrows). (**d**) A model for the cleavage of dsRNA with ADARs, TSN and hEndoV.

Tudor staphylococcal nuclease (TSN) is a subunit of the RNA-induced silencing complex^25^. It has been reported to promote I-RNase activity in various cell extracts (*Xenopus laevis* oocyte extracts, HeLa cell S100 extracts and HeLa nuclear extracts)^24^,^25^. We tested whether TSN could promote hEndoV activity in ssRNA containing inosine. The addition of TSN to our assays enhanced the cleavage activity of hEndoV, confirming that TSN was indeed a cofactor for the specific cleavage of inosine-containing RNA (Fig. 7a). As 3′,5′-deoxythymidine bisphosphate (pdTp) has been reported as an inhibitor of TSN for the cleavage of I-RNase^25^, we tested whether pdTp could inhibit hEndoV activity in ssRNA containing inosine. The addition of pdTp to the assays resulted in a reduction in cleavage of RNA substrates, suggesting that pdTp was an inhibitor of hEndoV (Fig. 7b). Taken together, these data suggest that hEndoV had endonuclease activity and acted as an I-RNase, specific to a certain structure of inosine-containing RNA.

Conversion of adenosine (A) residues to inosine (I) within dsRNA is catalysed by adenosine deaminases acting on RNA (ADARs)^20^. Although three ADARs (ADAR1, ADAR2 and ADAR3) have been described in mammals, only ADAR1 and ADAR2 have catalytic activity. TSN prefers the sites edited by ADAR2 over those edited by ADAR1 (ref. 26). We therefore examined whether hEndoV could cleave the predicted A to I sites edited by ADAR2 within dsRNA. Figure 7c shows that cleavage of 49-mer dsRNA was detected in the presence of ADAR2 and hEndoV, while little or no cleavage of the dsRNA was observed in the absence of ADAR2 and/or hEndoV. This finding indicated that hEndoV was capable of cutting dsRNA at the A to I edited sites, catalysed by the RNA-editing enzyme ADAR2.

## Discussion

In this study, we isolated and characterized a human *EndoV* gene and its resulting protein, which shared homology with the *nfi* gene. This protein could cleave a specific site in inosine-containing ssDNA substrates, acting with deoxyinosine 3′ endonuclease activity similar to that of eEndoV. Surprisingly, this enzyme could also cleave a specific site in inosine-containing ssRNA substrates. Analysis of hEndoV nuclease activity and DNA/RNA binding activity revealed that the enzyme preferred inosine-containing ssRNA substrates to inosine-containing ssDNA substrates. Immunochemistry and cell fractionation analyses showed that hEndoV was localized to the cytoplasm rather than the nuclei in cells. In addition, a local distorted single-strand structure of inosine-containing dsRNA and a second ribose 3′ to the lesion were required for nuclease activity of hEndoV. TSN, which stimulates I-RNase activity in cell extracts, promoted cleavage of hEndoV activity. Inosine formed by the catalytic activity of the A-to-I editing enzyme ADAR2 was a substrate for hEndoV. Taken together, these data indicated that hEndoV had endonuclease activity, as an I-RNase, specific to a certain structure of inosine-containing ssRNA and dsRNA. Although we cannot exclude the possibility that post-translational modifications of hEndoV, such as phosphorylation or ubiquitination, enhance its nuclease activity towards DNA containing deoxyinosine, we propose that the enzyme has a major role in removing inosine-containing RNA, which may adversely affect living cells.

Two fundamental mechanisms contribute to the generation of inosine in RNA. One mechanism is through A-to-I RNA editing by ADARs^20^. Two major isoforms of ADAR1 exist in mammalian cells: the p110 form is constitutively expressed, while the p150 form is induced by interferon^27^. p110 localization in the nucleus and p150 localization in both the nucleus and cytoplasm have been reported previously^27^, and more recent studies have suggested that both isoforms shuttle between the nucleus and cytoplasm^28^. RNA-regulated interaction of transportin-1 and exportin-5 with the dsRNA-binding domain regulates the nucleocytoplasmic shuttling of ADAR1 (ref. 28). ADAR2 is highly expressed in the brain and localizes to the nucleoli^29^ and cytoplasm^30^ in cells. Although we have shown that hEndoV was localized to the cytoplasm rather than the nuclei in cells under our experimental conditions, the localization of GFP-fusion hEndoV in the nucleoli and cytoplasm has been recently reported^16^. In both cases, hEndoV functions in locations where RNAs abound and are edited by ADARs, and not where DNA is found. Regarding the localization of the enzyme in cells, we realized the importance of detecting endogenous hEndoV by using anti-hEndoV antibodies as opposed to that by FLAG-tag or GFP fusion. However, because anti-hEndoV antibodies are not currently available for immunofluorescence microscopy, the precise subcellular localization of hEndoV is still unclear.

Editing occurs selectively within RNA and can result in codon changes, as inosine is interpreted as guanosine by the translation machinery. These ADARs predominantly catalyse RNA editing at specific sites in the dsRNA structure^17^,^20^ and are thought to hyperedit long dsRNA, which can result in up to 50% of the A residues being changed to I residues^31^,^32^. As hyperedited inosine-containing dsRNA is likely to have localized changes in its RNA structure due to the relative instability of IU pairs, ADARs generally diminish the double-strandedness of dsRNA. Although we have shown that hEndoV could cleave dsRNA treated by ADAR2, hEndoV can also cleave locally distorted ssRNA structures containing inosine. It may also cleave the dsRNA hyperedited by ADAR1. The functions of hyperedited long dsRNA are not fully understood. Interferons induce p150 to form ADAR1 and are cytokines with antiviral activity. Furthermore, viral infections in ADAR1-deficient HeLa cells result in enhanced apoptosis. Thus, hEndoV, together with TSN, may have a role in the antiviral response by removing the hyperedited long viral dsRNA genome that has undergone A-to-I editing (Fig. 7d)^18^,^24^,^33^,^34^. Moreover, it is possible that hEndoV may be involved in interdicting the RNA-silencing pathway. Long dsRNA is processed by the dsRNA-specific ribonuclease Dicer to produce small interference RNA, which is a key step in the RNA-silencing pathway. As dsRNA that is extensively edited by ADARs is known to be completely resistant to Dicer, hEndoV and/or TSN might participate in the degradation of this editing dsRNA, consequently reducing the expression of small interference RNA (Fig. 7d)^20^,^35^.

The other mechanism that produces inosine in RNA is spontaneous and incidental modification by hydrolysis, nitrosative chemistry and incorporation of ITP^1^,^2^,^3^. Elevated temperatures at pH 7.4 induced the conversion of adenine to hypoxanthine in ssDNA, and this condition will also generate hypoxanthine in mRNA. Nitrosative stress caused by increases in nitric oxide-derived nitrous anhydride during inflammation produces hypoxanthine from adenine. Moreover, unexpected incorporation of ITP into mRNA is brought about by RNA polymerase during transcription. Although ITP is removed by an inosine triphosphatase from the cellular nucleotide pool, ITP is formed because of defects in purine nucleotide metabolism. Inosines generated in mRNA via this process are thought to be potentially mutagenic, as inosine is recognized as guanine during translation, thereby generating mutant proteins that potentially inhibit cell viability^36^,^37^. Moreover, A-to-I editing of mRNA by ADARs is a highly regulated mechanism controlling gene expression and gene integrity^17^,^20^. Inosines in mRNA could be interpreted as inappropriate editing. Additionally, inosines in rRNA and tRNA could affect translational fidelity and efficiency. Therefore, removing unexpected inosines in RNA is an important cellular mechanism; inosine-containing RNA may be removed from living cells by hEndoV.

Deoxyinosine in DNA is also mutagenic because deoxyinosine is recognized as deoxyguanosine by replicative DNA polymerases; the lesions should be removed from DNA before replication. According to the results of *E. coli* genetic studies, the deoxyinosine 3′ endonuclease eEndoV is a repair enzyme that removes deoxyinosine in AER. The absence of eEndoV induces A:T to G:C transitions in response to nitrous acid treatment, generating deoxyinosine in DNA. Previous studies have implicated hEndoV in the AER mechanism^8^,^15^. Additionally, mammalian EndoV could complement the mutagenesis phenotype in the *E. coli nfi* mutant. However, we think that these proteins may be highly overexpressed in that system. Regarding the biochemical results in mammalians, we and other group could not show significant deoxyinosine 3′ endonuclease activity of hEndoV in dsDNA substrates^16^. Using micromolar concentrations of mEndoV and nanomolar concentrations of the annealed dsDNA substrate for hEndoV, these enzymes exhibited cleavage of the dsDNA substrate containing deoxyinosine. Even under the same experimental conditions in which mammalian EndoV cleaved dsDNA, the enzyme does not exhibit more efficient removal of deoxyinosine in dsDNA than in ssDNA^8^,^15^. Although we cannot exclude the possibility that post-translational modifications of hEndoV enhance its nuclease activity towards DNA, human alkyladenine DNA glycosylase may have a major role in this repair process, as the enzyme catalyses the hydrolysis of N-glycosidic bonds to release damaged bases, including hypoxanthine^38^,^39^. Together, we conclude that hEndoV is a novel RNA-modifying enzyme that has a central role in removing inosine-containing RNA.

## Methods

### DNA and RNA substrates

Thirty-mer DNA substrates and 21-mer RNA substrates containing hypoxanthine were synthesized at FASMAC (Kanagawa, Japan) and purified using HPLC. Twenty-mer RNA substrates containing IUII or UUIU sequences and 49-mer dsRNA substrates were synthesized on an Applied Biosystems 3400 DNA synthesizer (Applied Biosystems, Carlsbad, CA) by using phosphoramidite building blocks purchased from Glen Research (Sterling, VA) and were purified using HPLC. The oligonucleotides (Supplementary Table S1) were 5′-phosphorylated using (γ-^32^P)-ATP (PerkinElmer Life Sciences, Waltham, MA) and T4 phosphoramidite kinase (TaKaRa, Shiga, Japan). Unincorporated nucleotides were removed using MicroSpin G-25 columns (GE Healthcare, Waukesha, WI).

### Proteins

hEndoV and mutants D52A, Y91A and E100A were purified as follows. hEndoV cDNA (FLJ35220) was amplified from total human dermal fibroblast RNA using a OneStep RT-PCR kit (Qiagen, Valencia, CA) with specific primers (5′-CAGGAATTCCCATGGCCCTGGAGGCGGCGGGA-3′ and 5′-CGAGTCGACTCAACAAAGTGCTGAGGACTC-3′) and cloned into pGEX-6p-2 (GE Healthcare). All the constructs were checked by DNA sequencing. The proteins were then expressed in *E. coli* Rosseta 2 (Novagen), purified using DEAE Sepharose FF, Glutathione Sepharose 4 Fast Flow and HiTrap Heparin HP columns (GE Healthcare), as recommended by the manufacturer. Fractions containing proteins from the HiTrap Heparin HP column were eluted in 300 mM KCl, 20 mM Tris-HCl buffer (pH 8.0), 10% glycerol, 0.1 mM EDTA and 1 mM DTT. All the proteins were analysed by SDS–PAGE and visualized by Coomassie blue staining. The eEndoV was obtained from New England Biolabs. Protein concentrations were measured using a Bio-Rad Protein Assay kit (Bio-Rad, Hercules, CA). Although we used GST-tag enzymes, GST tag did not disturb the hEndoV function (Supplementary Fig. S7). The GST tag was cleaved using PreScission Protease (GE Healthcare), according to the manufacturer’s instructions.

### Cleavage assays

Standard cleavage reaction mixtures (5 μl) contained 0.2 nM of ^32^P-labelled substrate and the indicated amount of hEndoV or mutant hEndoVs (D52A, Y91A or E100A) in a reaction buffer containing 50 mM potassium acetate, 20 mM Tris-acetate (pH 7.9), 10 mM magnesium acetate and 1 mM DTT. The reactions were incubated at 37 °C for 30 min and then terminated with sequencing stop buffer (5 μl) containing 98% deionized formamide, 20 mM EDTA, 0.025% bromophenol blue and 0.025% xylene cyanol. Fragments were separated on a 12.5% denaturing polyacrylamide gel. The dried gel was analysed using a Fuji FLA-7000 phosphorimager (Fujifilm, Tokyo, Japan).

### Electrophoretic mobility shift assays

Standard binding reaction mixtures (5 μl) contained 0.2 nM ^32^P-labelled substrate and the indicated amount of hEndoV or mutant hEndoVs (D52A, Y91A or E100A) in a reaction buffer containing 10 mM HEPES-KOH (pH 8.0), 1 mM DTT, 2% glycerol and 5 mM CaCl_2_. Reactions were incubated at 4 °C for 30 min, and loading buffer (0.5 μl) containing 50% glycerol, 0.5% bromophenol blue, and 0.5% xylene cyanol was subsequently added.

Samples were separated on a nondenaturing 8% polyacrylamide gel. The dried gel was analysed using a Fuji FLA-7000 phosphorimager (Fujifilm).

### Subcellular fractionation

HEK293 cells (Invitrogen, Carlsbad, CA) stably expressing C-terminally FLAG-V5-6xHis-tagged hEndoV were established using a pcDNA5/FRT/V5-His TOPO TA Expression Kit (Invitrogen), according to the manufacturer’s instructions. A subcellular protein fractionation kit (Thermo Fisher Scientific, Waltham, MA) was used to fractionate proteins into nuclear and cytoplasmic fractions using the manufacturer’s protocol. The whole-cell extract was saved separately. Samples were boiled with 2 × SDS sample loading buffer before analysis.

### Immunofluorescence microscopy

Cells grown on coverslips were washed with PBS and fixed with 4% formaldehyde in PBS at room temperature (RT) for 20 min. The cells were washed three times with PBS and then incubated in PBS containing 0.1% Triton X-100 at RT for 30 min. The samples were blocked for 30 min with Blocking One buffer (Nacalai Tesque, Kyoto, Japan) and incubated with anti-V5 antibodies (Invitrogen, dilution 1:500) at RT for 1 h. The samples were subsequently washed and incubated with Alexa Fluor-conjugated antibodies (Molecular Probes, dilution 1:200) at RT for 1 h. Samples were mounted using VECTASHIELD Mounting medium (Vector Laboratories, Burlingame, CA) that contained 4',6-diamidino-2-phenylindole dihydrochloride and were examined under an IX71 fluorescence microscope system (Olympus, Center Valley, PA).

## Author contributions

Y.M., T.S. and N.N. performed experimental work. K.N., S.I. and I.K. designed the research. I.K. wrote the manuscript.

## Additional information

**How to cite this article:** Morita, Y. *et al.* Human endonuclease V is a ribonuclease specific for inosine-containing RNA. *Nat. Commun.* 4:2273 doi: 10.1038/ncomms3273 (2013)

## Supplementary Material

Supplementary Information Supplementary Figures S1-S7 and Supplementary Table S1
