# Supplementary material for: Human endonuclease V is a ribonuclease specific for inosine-containing RNA
Source: Nat Commun. 2013 Aug 5;4:2273. doi: 10.1038/ncomms3273 (PMC3741642; doi:10.1038/ncomms3273)
Supplement: Supplementary Information — Supplementary Figures S1-S7 and Supplementary Table S1 [file ncomms3273-s1.pdf]

Supplementary Information

Human endonuclease V is a ribonuclease specific for inosine-containing RNA

Yoko Morita[1], Toshihiro Shibutani[1], Nozomi Nakanishi[1], Kazuko Nishikura[2], Shigenori Iwai[1], Isao Kuraoka[1*]

[1]Graduate School of Engineering Science, Osaka University, 1-3 Machikaneyama, Toyonaka, Osaka 560-8531, Japan. [2]The Wistar Institute, 3601 Spruce Street, Philadelphia, PA 19104, USA.

***Corresponding author:** Isao Kuraoka, Graduate School of Engineering Science, Osaka University, 1-3 Machikaneyama, Toyonaka, Osaka 560-8531, Japan. Tel: +81-6-6850-6240, Fax: +81-6-6850-6240, E-mail: kuraoka@chem.es.osaka-u.ac.jp

Short Title: hEndoV as an I-RNase

Keywords: Deamination, DNA repair enzyme, ribonuclease, RNA editing

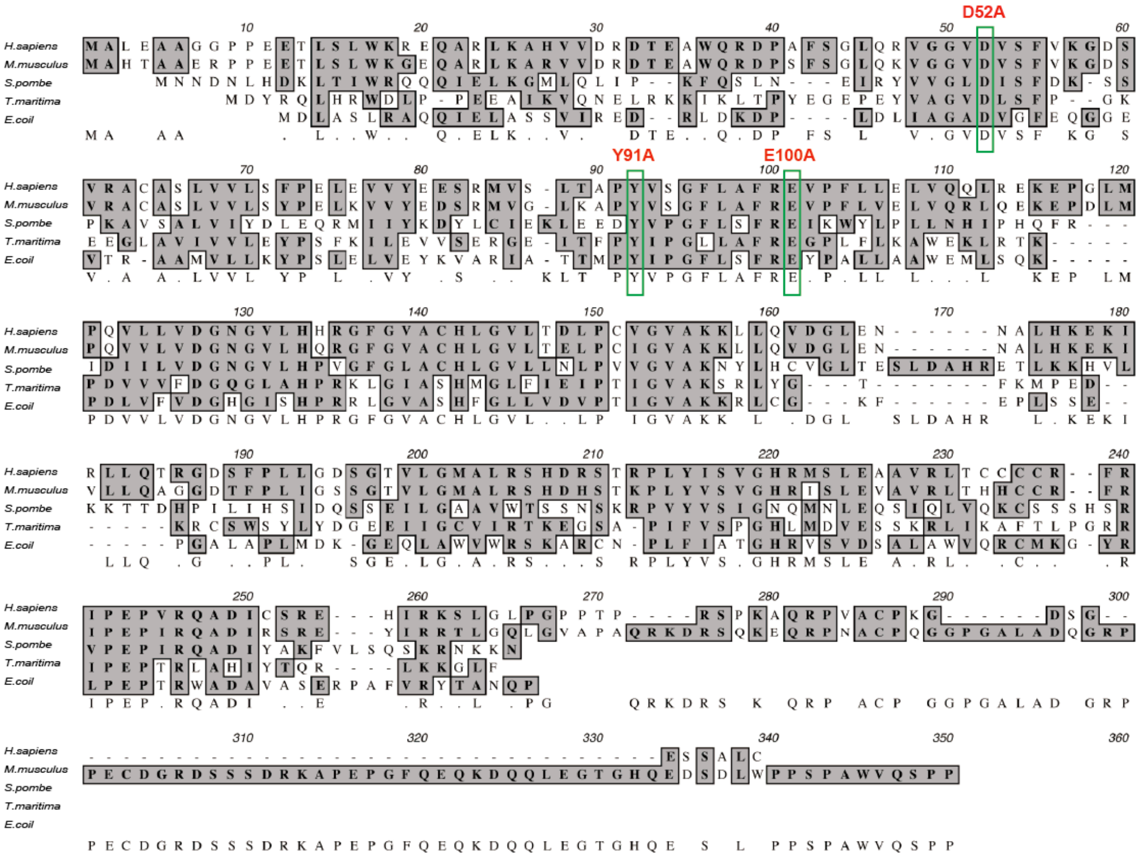

Supplementary Fig. S1

Supplementary Fig. S1. Human endonuclease V (hEndoV).

Alignment of the representative amino acid sequences of EndoV from *Escherichia coli* (NP_290630), *Thermotoga maritima* (NP_229661), *Schizosaccharomyces pombe* (NP_594332), *Mus musculus* (NP_001158108), and humans (NP_775898). Identical and similar residues among the enzymes are indicated in gray.

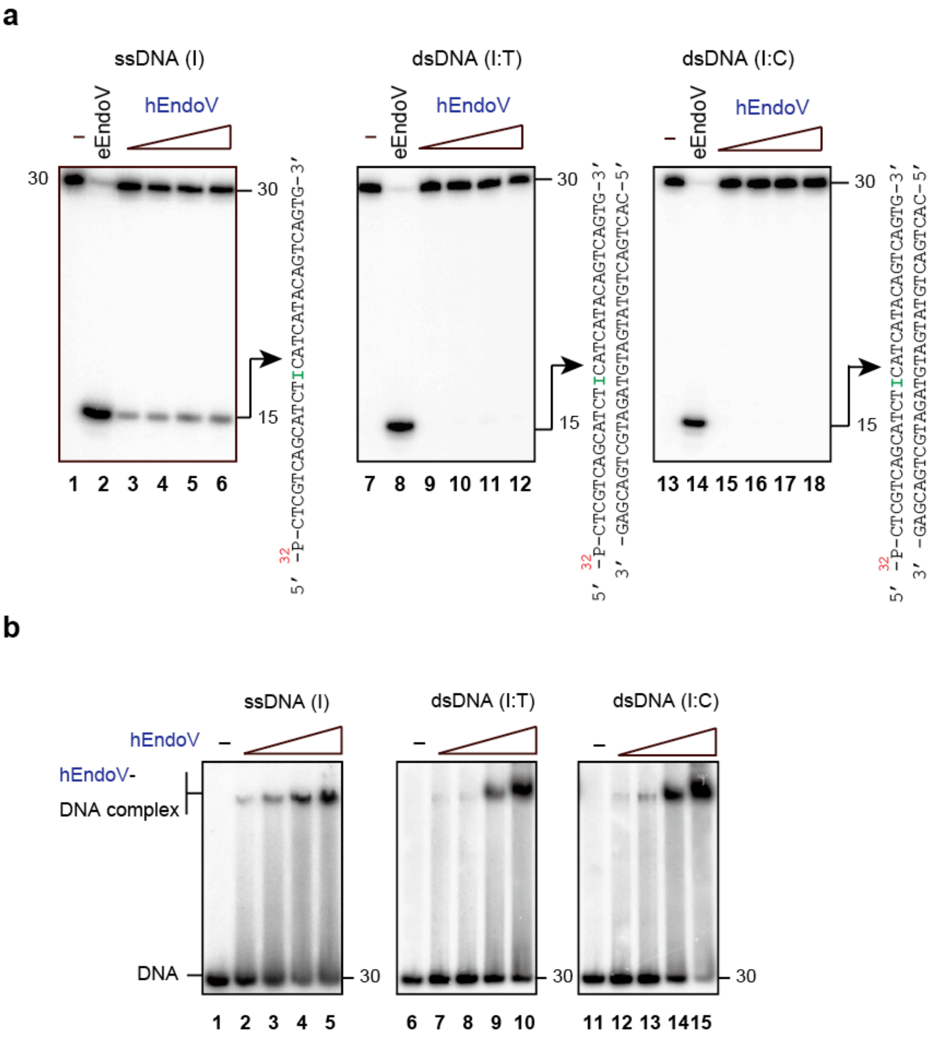

Supplementary Fig. S2

Supplementary Fig. S2. Properties of hEndoV with respect to inosine-containing DNA. (a) ^{32}P-labeled 30-mer ssDNA containing deoxyinosine (lanes 1–6), ^{32}P-labeled 30-mer dsDNA containing deoxyinosine paired with deoxythymidine (lanes 7–12), or ^{32}P-labeled 30-mer dsDNA containing deoxyinosine paired with deoxycytidine (lanes 13–18) was incubated with increasing concentrations of hEndoV (0.125, 0.25, 0.5, and 1.0 µM in each group of 4 lanes) or eEndoV (5 nM). A 30-mer ssDNA or dsDNA (right panel in each figure) molecule was cleaved at the indicated positions (arrows, 15-mer). (b) ^{32}P-labeled 30-mer ssDNA or dsDNA shown in (a) was incubated at 4°C for 30 min with increasing concentrations of hEndoV (0, 12.5, 25, 50, and 100 nM in each group of 5 lanes). Free and bound fractions were separated on a nondenaturing 8% polyacrylamide gel.

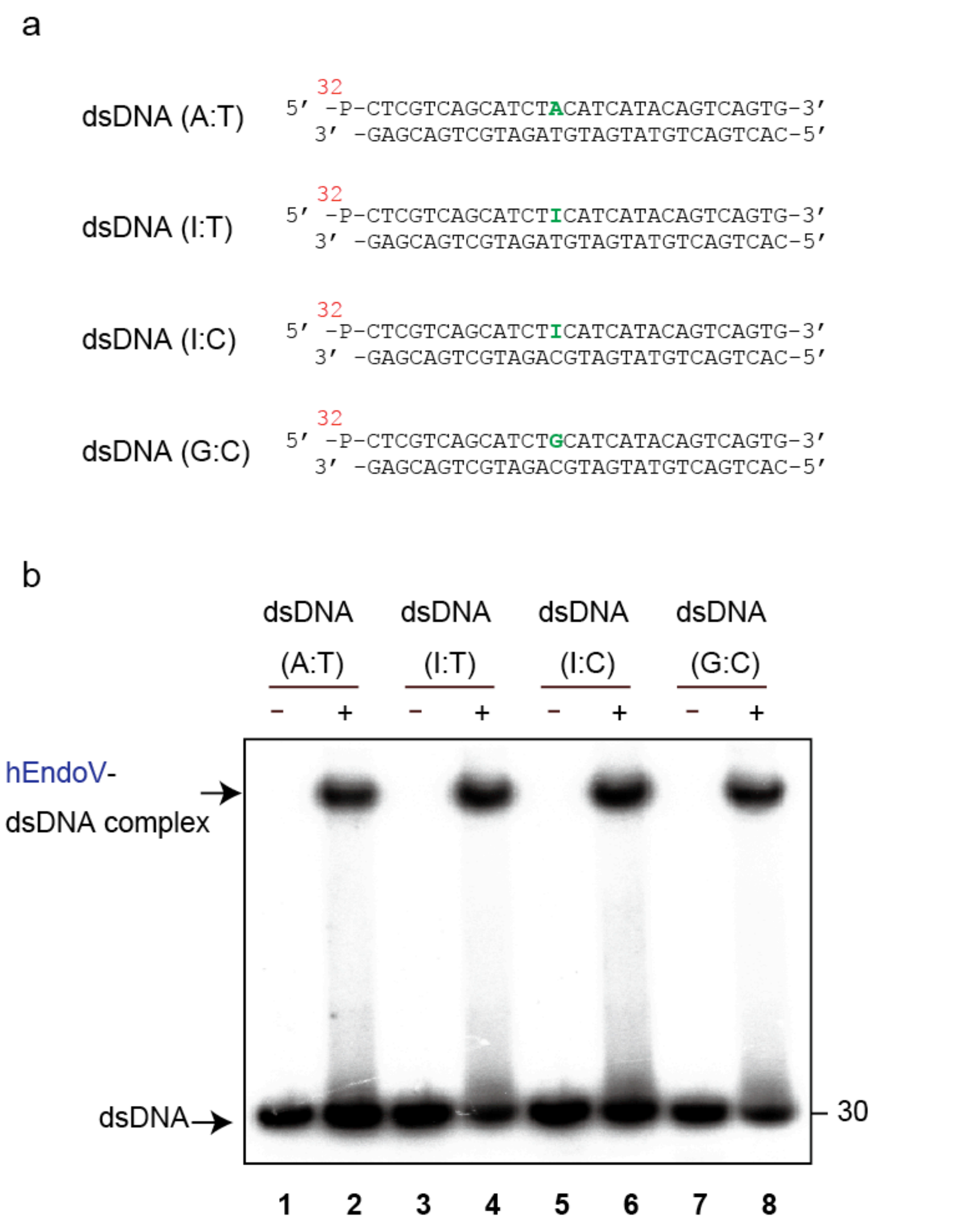

Supplementary Fig. S3

Supplementary Fig. S3. The binding properties of hEndoV for inosine-containing dsDNA. (a) A series of dsDNA substrates containing deoxyadenosine, deoxyinosine, or deoxyguanosine. (b) The ^{32}P-labeled 30-mer dsDNA shown in (a) was incubated at 4°C for 30 min with (+) or without (-) 50 nM hEndoV. Free and bound fractions were separated on a nondenaturing 8% polyacrylamide gel.

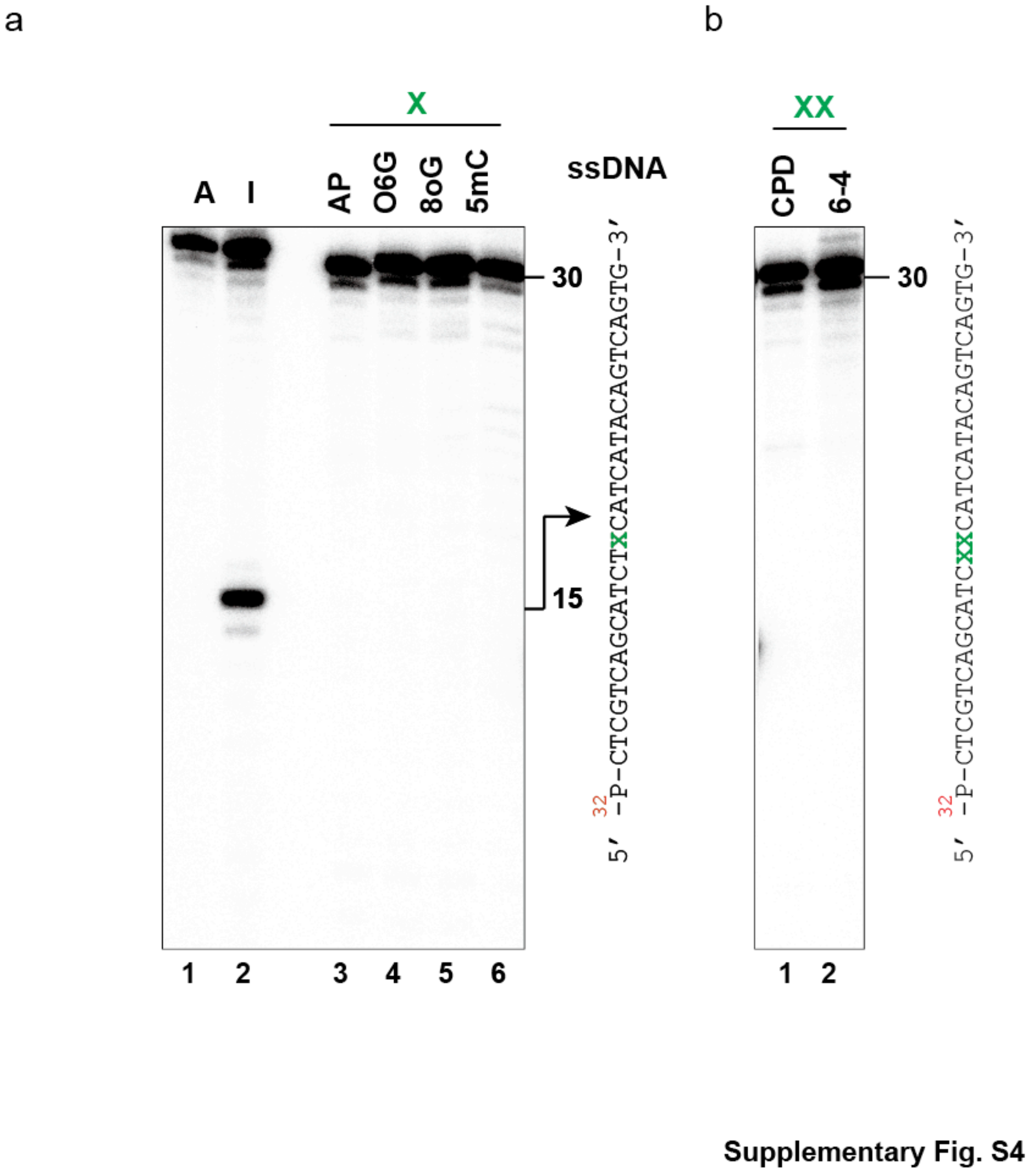

Supplementary Fig. S4. Properties of hEndoV with respect to inosine-containing DNA. (a) ^{32}P-labeled 30-mer ssDNA containing deoxyadenosine (lane 1), deoxyinosine (lane 2), AP site (lane 3), *O*^{6}-methlyguanine (lane 4), 8-oxoguanine (lane 5), or 5-methlycytosine (lane 6) in position X (*right panel*) was incubated with hEndoV (1 µM). A 30-mer ssDNA (*right panel*) was cleaved at the indicated positions (arrows, 15-mer). (b) ^{32}P-labeled 30-mer ssDNA containing *cis*-syn cyclobutane pyrimidine dimers (lane 1) or pyrimidine (6-4) pyrimidone photoproducts (lane 2) in position XX (*right panel*) was incubated with hEndoV (1 µM).

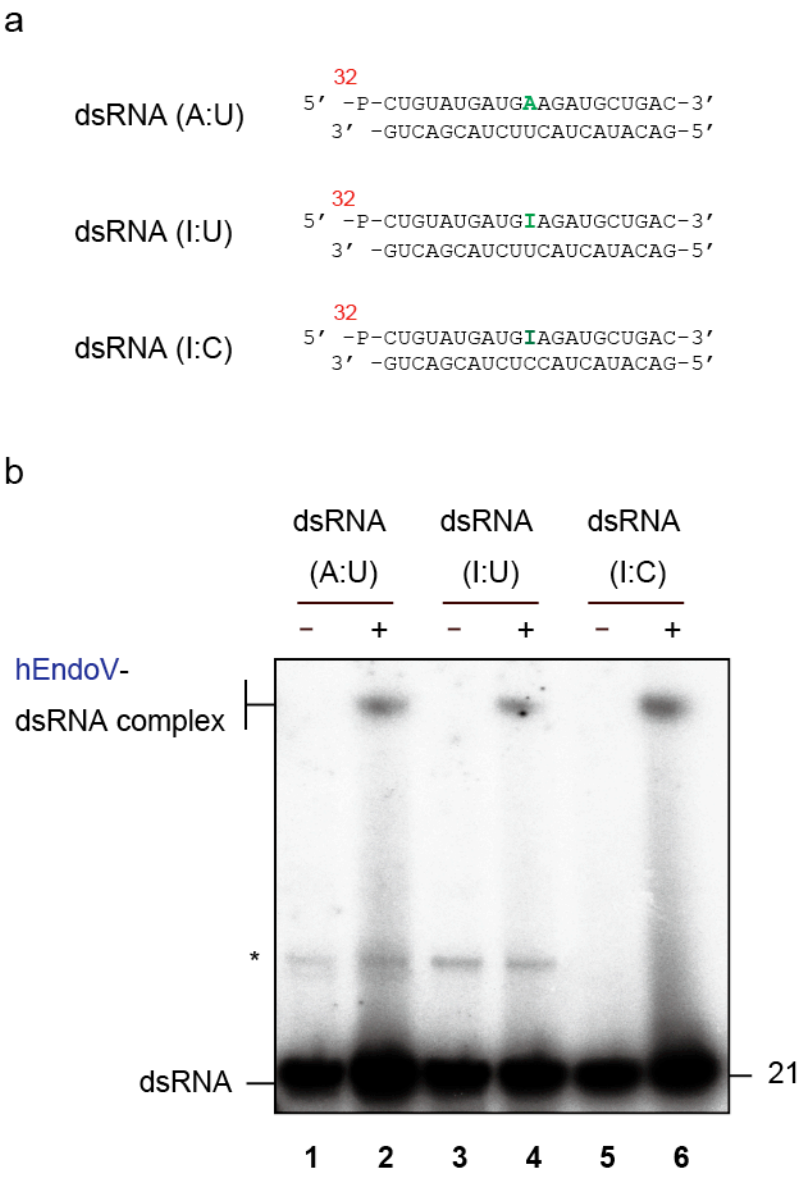

Supplementary Fig. S5

Supplementary Fig. S5. Binding properties of hEndoV for inosine-containing dsRNA. (a) A series of RNA substrates containing adenine or inosine in dsRNA. (b) ^{32}P-labeled 21-mer dsRNA shown in (a) was incubated at 4°C for 30 min with (+) or without (-) hEndoV (20 nM). The band indicated with an asterisk is a nonspecific band. Free and bound fractions were separated on a nondenaturing 8% polyacrylamide gel.

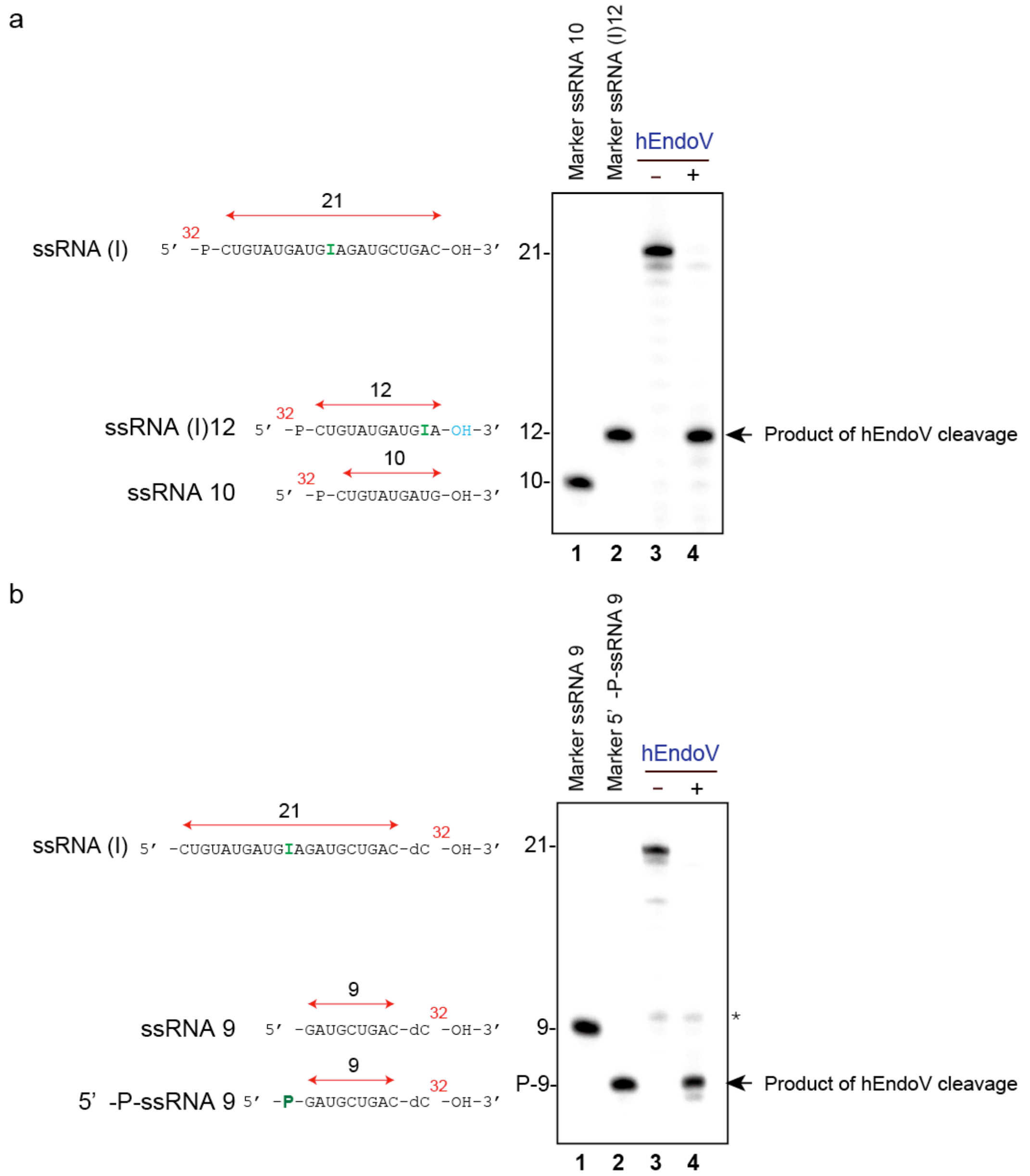

Supplementary Fig. S6. The nuclease properties of hEndoV for inosine-containing ssRNA. (a) 5′-^{32}P-labeled 21-mer ssRNA containing inosine was incubated at 37°C for 30 min without (-) or with (+) 20 nM hEndoV (lanes 3 and 4). Size markers, 5′-^{32}P-labeled ssRNA 10-mer oligo (lane 1) and 5′-P-labeled ssRNA 12-mer oligo containing inosine (lane 2). (b) 3′-^{32}P-labeled 21-mer ssRNA containing inosine was incubated at 37°C for 30 min without (-) or with (+) 20 nM hEndoV (lanes 3 and 4). Size markers, 3′-^{32}P-labeled ssRNA oligo 9-mer (lane 1) and 3′-P-labeled ssRNA oligo 9-mer containing phosphate at the 5′ position (lane 2). The band indicated with an asterisk is a nonspecific band. Fragments were separated on a 15% denaturing polyacrylamide gel.

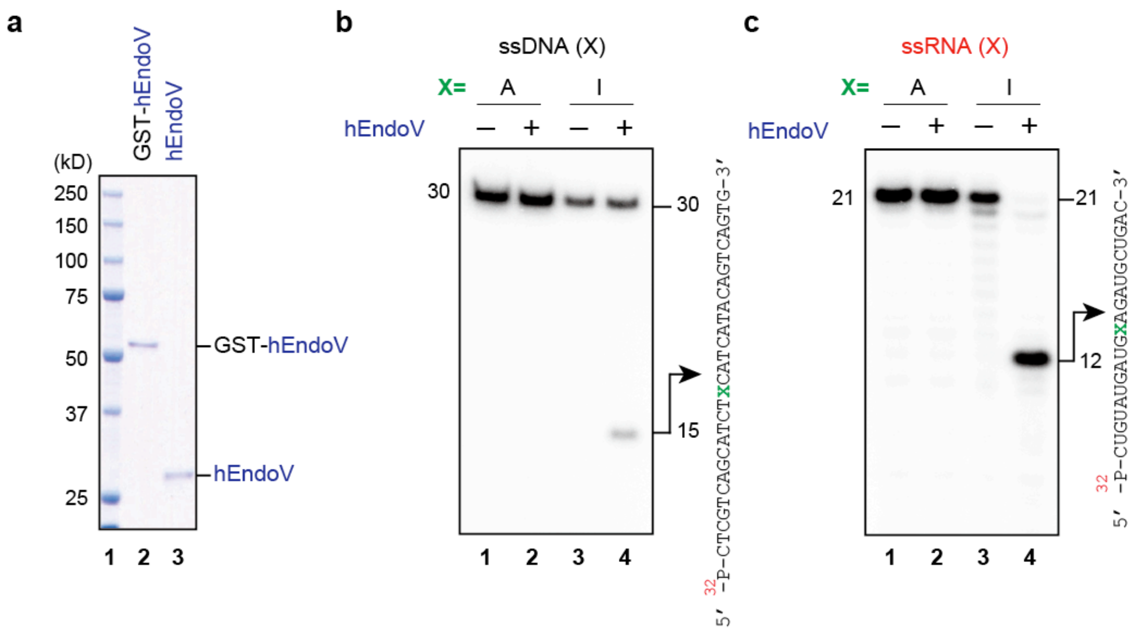

Supplementary Fig. S7

Supplementary Fig. S7. Nuclease properties of hEndoV without GST-tag.

(a) Purified proteins were subjected to SDS-PAGE on 10% gels, and the proteins were visualized by staining with CBB. Lane 1, marker; lane 2, GST-hEndoV; lane 3, hEndoV. (b) 5′-^{32}P-labeled 30-mer ssDNA containing deoxyadenine or deoxyinosine was incubated at 37°C for 30 min without (-, lanes 1 and 3) or with (+, lanes 2 and 4) 300 nM hEndoV. (c) 5′-^{32}P-labeled 21-mer ssRNA containing adenine or inosine was incubated at 37°C for 30 min without (-, lanes 1 and 3) or with (+, lanes 2 and 4) 30 nM hEndoV.
